# N-acetyl-L-cysteine (NAC) delays post-ovulatory oocyte aging in mouse

**DOI:** 10.18632/aging.101898

**Published:** 2019-04-12

**Authors:** Yue Wang, Li Li, Li-Hua Fan, Ying Jing, Jian Li, Ying-Chun Ouyang, Zhen-Bo Wang, Yi Hou, Qing-Yuan Sun

**Affiliations:** 1 State Key Laboratory of Stem Cell and Reproductive Biology, Institute of Zoology, Chinese Academy of Sciences, Beijing, China; 2 University of Chinese Academy of Sciences, Beijing, China; 3 Department of Reproductive Medicine, Peking University Shenzhen Hospital, Shenzhen Peking University-The Hong Kong University of Science and Technology Medical Center, Shenzhen, China

**Keywords:** post-ovulatory aging, oocyte, mitochondria, ROS

## Abstract

The quality of post-ovulatory oocytes decreases with aging. In this study, we aimed to investigate the effects of N-acetyl-L-cysteine (NAC), a broadly used antioxidant, on oocyte quality in mouse post-ovulatory oocyte aging in vitro. NAC at 0.6mM concentration was added to culture medium (M2), and the quality of oocytes was analyzed at 6h, 12h, 18h and 24h of culture. We found that the frequency of spindle defects decreased in NAC-treated oocytes compared to those without NAC treatment. NAC treatment significantly decreased abnormal distribution of cortical granules (CGs) in oocytes during aging for 18h and 24h. Decreased intracellular reactive oxygen species (ROS) was also observed. Increased intracellular ATP levels and decreased abnormal distribution of mitochondria could be observed with NAC supplementation during post-ovulatory oocyte aging in vitro. These results indicate that NAC will maintain the quality of oocytes, and delay post-ovulatory oocyte aging as studied in the mouse.

## INTRODUCTION

In the fetal ovary, oocytes are derived from primordial germ cells (PGCs), and they are arrested at the diplotene (germinal vesicle, GV) stage of the first meiotic division until puberty takes place [1–3]. Upon luteinizing hormone (LH) surge stimulation, fully-grown oocytes develop and complete the first meiotic division with the first polar body being extruded. Oocytes finally arrest at the metaphase of the second meiotic division until fertilization takes place. If no fertilization occurs, oocytes undergo a time-dependent deterioration in quality, referred to as postovulatory aging [4, 5].

Postovulatory aged oocytes display decreased fertilization rates [6, 7], increased chromosome and spindle anomalies [6, 9, 10], partial exocytosis of cortical granules (CG) [8, 10], zona hardening [6, 11], and increased susceptibility to activating stimuli [6, 12]. Additionally, aged oocytes exhibit mitochondrial dysfunction [6, 13]. Mitochondria are the most numerous organelles in oocytes and they are crucially affected during the process of oocyte aging [14]. The distribution of mitochondria is a highly dynamic process during oocyte maturation and early embryonic development [6]. It has previously been shown that mitochondria are symmetrically distributed in the cytoplasm, displaying a concentrated distribution around the MII spindle in mature oocytes; thus, the abnormal distribution of mitochondria is a marker of aged oocytes [6, 15]. As the most important function of mitochondria is to generate energy in the form of adenosine triphosphate (ATP), mitochondrial dysfunction leads to decreased ATP production, and relatively low ATP content was observed in aged oocytes [16, 17]. Endogenous reactive oxygen species (ROS) such as superoxide and H_2_O_2_ are produced continuously in mitochondria along the electron transport chain [8]. Once the ROS level produced by dysfunctional mitochondria exceeds the antioxidant defense capacity, oxidative stress is induced, which can lead to oocyte dysfunctions or apoptosis [16, 18].

To increase fertilization and the embryo’s developmental potential, maintaining the quality of oocytes is essential. In order to reduce oxidative stress and potentially reduce ROS-induced damage, antioxidant supplementation is widely used. The antioxidant N-acetyl-L-cysteine (NAC), a reactive oxygen species (ROS) scavenger, has been shown to effectively prevent smoke-induced developmental defects and telomere dysfunction [19]. Likewise, long-term dietary supplementation with the antioxidant NAC decreases oxidative stress in cells from ataxia telangiectasia (AT) patients and significantly increases the lifespan, reducing both the incidence and multiplicity of lymphoma in *Atm* deficient mice [20]. Moreover, NAC can be safely used in humans, and its long-term usage in humans is beneficial during radiation therapy to prevent radiation-mediated genotoxicity [21]. Results also showed that treatment of mice for 2 months with NAC could improve the quality of fertilized eggs and early embryo development in vivo [22]. Supplementation with 1.5 mM NAC to the oocyte maturation medium increased the percentage of viable embryos that reach the blastocyst stage of development [23]. Based on these data, we hypothesized that NAC supplementation may have similar effects on delaying oocyte aging. The antioxidant N-acetyl-L-cysteine (NAC), working as a reactive oxygen species (ROS) scavenger, has already been widely used in medicine for several years, especially as potent drug for Acetaminophen poisoning. On a widely used basis, of NAC has been shown to have little toxicity to the human body.

In this study, we investigated whether NAC can effectively prevent post-ovulatory oocyte aging by supplementation during in vitro culture. We hypothesized that timed treatment of mature MII oocytes with NAC would delay the post-ovulatory quality deterioration of oocyte aging in vitro.

## RESULTS

### Treatment with NAC delays formation of spindle anomalies during post-ovulatory oocyte aging in vitro

**Figure 1:**
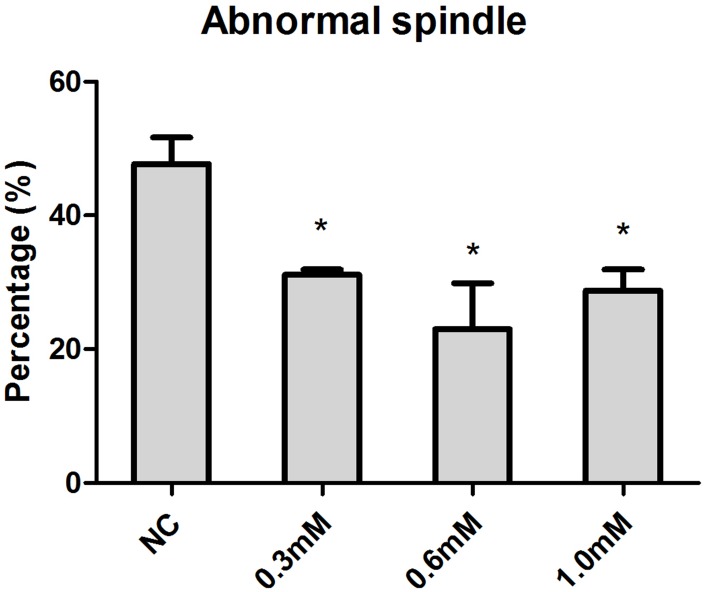
**Concentration-dependent effect of NAC on spindle morphology in post-ovulatory oocytes aging for 24h in vitro.** Oocytes cultured with different concentrations of NAC for 24h in vitro were collected for immunofluorescence analysis. Oocytes with normal spindles and those showing abnormal spindles were counted to calculate the percentage of abnormal spindles. X-axis represents the concentrations of NAC. NC, the oocyte without NAC treatment. Y-axis represents the percentages of oocytes with abnormal morphological spindle. Data are expressed as mean ± SEM of at least 3 independent experiments, and 6 superovulated mice were killed to obtain a minimum of 40 oocytes for each experiment. Star represents mean differ 0.01< p < 0.05.

Increasing concentrations (0.3mM, 0.6mM, 1.0mM) of NAC were supplied to culture medium (M2) for in vitro oocyte culture to test whether NAC treatment could delay aging-induced changes in spindle anomalies. Oocytes aged in vitro were collected after 24h of culture for immunofluorescence analysis. The results showed that fresh oocytes displayed bipolar spindles with focused poles. Various formations of severely abnormal spindles were found in aged oocytes, including elongated spindles and spindles with abnormal poles, with the major spindle defect being spindles with no apparent poles. We found significant differences in oocytes treated with 0.3mM, 0.6mM, and 1.0 mM NAC displaying a lower rate of abnormal spindles compared to oocytes without NAC treatment (Figure 1), suggesting that NAC delayed post-ovulatory oocyte aging in vitro. Due to fewer numbers of spindle anomalies caused by 0.6mM NAC, we used this concentration for all subsequent experiments.

**Figure 2:**
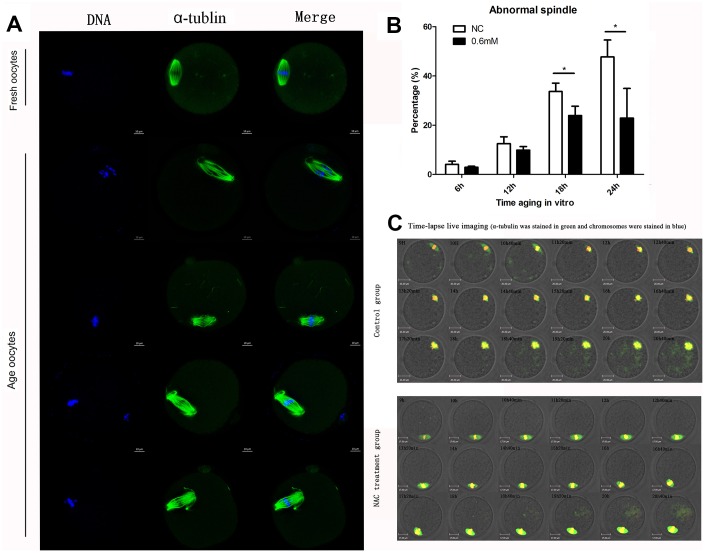
**Treatment with NAC delays the changes in spindle anomalies of post-ovulatory oocyte aging in vitro.** (**A**) Different morphological structures of spindles appeared in fresh and aged oocytes. Meiotic spindles in oocytes were stained with α-tubulin (green) and chromosomes were stained with Hoechst 33342 (blue). (**B**) Percentages of abnormal spindles in oocytes. Oocytes containing normal spindles and those with abnormal spindles were counted to calculate the percentage of abnormal spindles. NC, oocytes treated without NAC. 0.6mM, oocytes treated with 0.6mM NAC. Data are expressed as mean ± SEM of at least 3 independent experiments, and 6 superovulated mice were killed to obtain a minimum of 40 oocytes for each experiment. Data are expressed as mean ± SEM. Star represents mean difference, 0.01< p < 0.05. (**C**) Control or NAC treated oocytes, which were microinjected with MAP4-eGFP mRNA and H2B-mCherry mRNA, and visualized by time-lapse live-cell imaging. The spindles are marked in green, and chromosomes are marked in red. Independent replicates were conducted with a minimum of 20 oocytes.

We then investigated the morphological alterations of spindles during oocyte aging for different times with or without NAC treatment. Confocal microscopy showed that various morphological spindle structures were formed during post-ovulatory oocyte aging (Figure 2A). The percentages of abnormal spindles were analyzed at 6h, 12h, 18h, and 24h of postovulatory oocyte aging in vitro. Oocytes cultured for 12h, 18h and 24h showed increased percentages of abnormal spindles compared to fresher oocytes which were cultured for 6h, in both NAC-treated and control group. When MII oocytes were exposed to 0.6mM NAC, the percentage of abnormal spindles observed at 18h or 24h was significantly lower compared to that in control untreated oocytes, indicating that NAC treatment could delay the changes in spindle anomalies at 18h and 24h of post-ovulatory oocyte aging in vitro (Figure 2B). There was no significant difference between the NAC treatment group and control group at 12h during post-ovulatory oocyte aging in vitro.

Next, live cell imaging was performed to analyze the dynamic changes of spindles after treatment with or without NAC. In control aging oocytes, spindles gradually lose poles. At around 14h and 40 minutes of live cell imaging, spindles without one pole were observed. At 20h and 40 minutes, both poles disappeared. In contrast, normal morphological spindles were observed at 14h and 40 minutes when oocytes were treated with NAC. At 20h and 40 minutes of live cell imaging, the two poles could still be seen in the spindles (Figure 2C). The results showed that NAC treatment could delay the changes in spindle anomalies during post-ovulatory oocyte aging in vitro.

### NAC administration maintains the normal distribution of CGs

**Figure 3:**
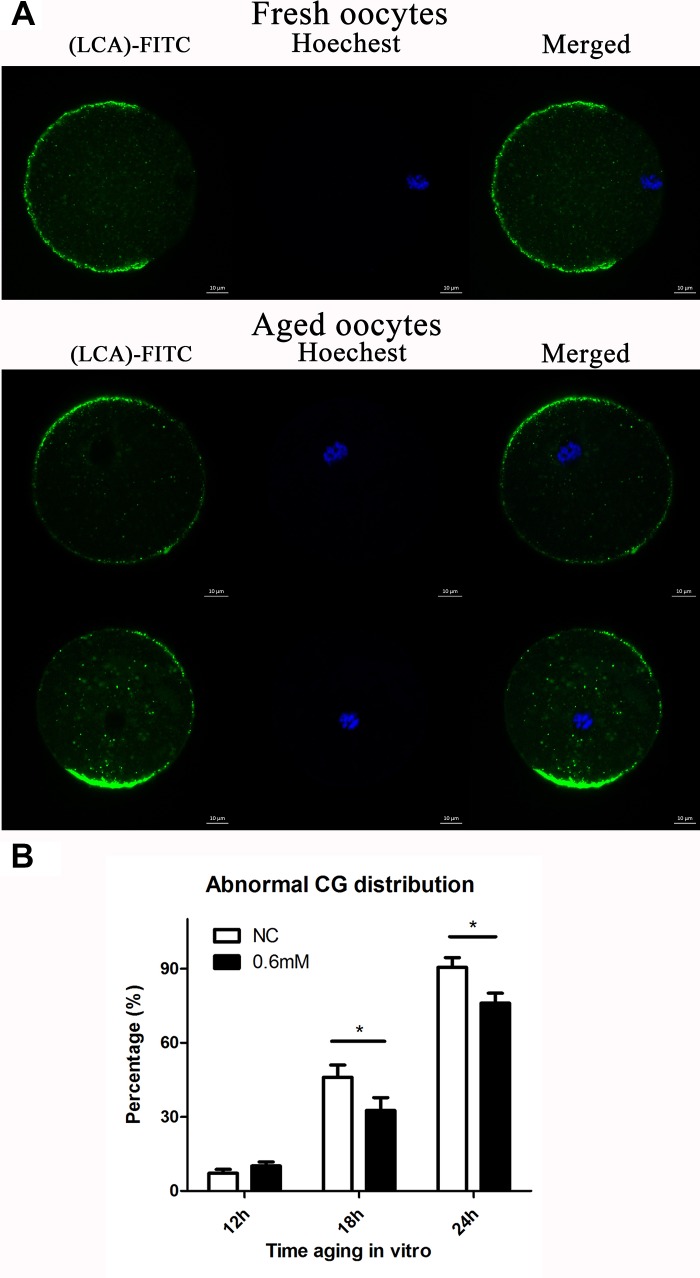
**NAC administration maintains the normal distribution of CGs.** (**A**) Different patterns of CG distribution in fresh and aged oocytes. CGs in oocytes were stained with lens culinaris (LCA)-FITC (fluorescein isothiocyanate: green) and chromosomes were stained with Hoechst 33342 (blue). The total number of oocytes in each experiment and the number of oocytes which showed abnormal CG distribution were counted to calculate the percentage of abnormal CG distribution in oocytes. (**B**) Percentages of abnormal distribution of CGs in oocytes. NC, oocytes treated without NAC. 0.6mM, oocytes treated with 0.6mM NAC. Data are expressed as mean ± SEM of at least 3 independent experiments, and 6 superovulated mice were killed to obtain a minimum of 40 oocytes for each experiment.

It has previously been shown that post-ovulatory oocyte aging causes morphological changes in the spindle, as well as abnormal distribution in CGs. We investigated the distribution of CGs during post-ovulatory oocyte aging in vitro with or without NAC treatment. Confocal microscopy showed that different distribution patterns of CGs appeared during post-ovulatory oocyte aging in vitro. We determined that oocytes displayed a layer of CGs beneath the oolemma and a distinct CG-free domain near the area of the spindle apparatus that we called normal distribution in fresh oocytes. On the other hand, during post-ovulatory oocyte aging, CG congregation occurred. We found two major types of abnormal distribution: type I, all CGs moved to the cortex of oocytes without leaving CG-free domains; Type II, CGs moved to CG-free domains near the chromosomes (Figure 3A), and the percentage of abnormal distribution of CGs clearly increased obviously at 18h and 24h of in vitro culture in contrast to fresh oocytes. However, compared with control oocytes, the oocytes treated with 0.6mM NAC showed a lower percentage of abnormal CG distribution at 18h and 24h (Figure 3B), indicating its protective effect on post-ovulatory oocyte aging in vitro. However, no significant difference was observed between the NAC supplementation group and the control group at 12h of culture in vitro.

### Supplementation of NAC decreases the intra-cellular ROS level

**Figure 4:**
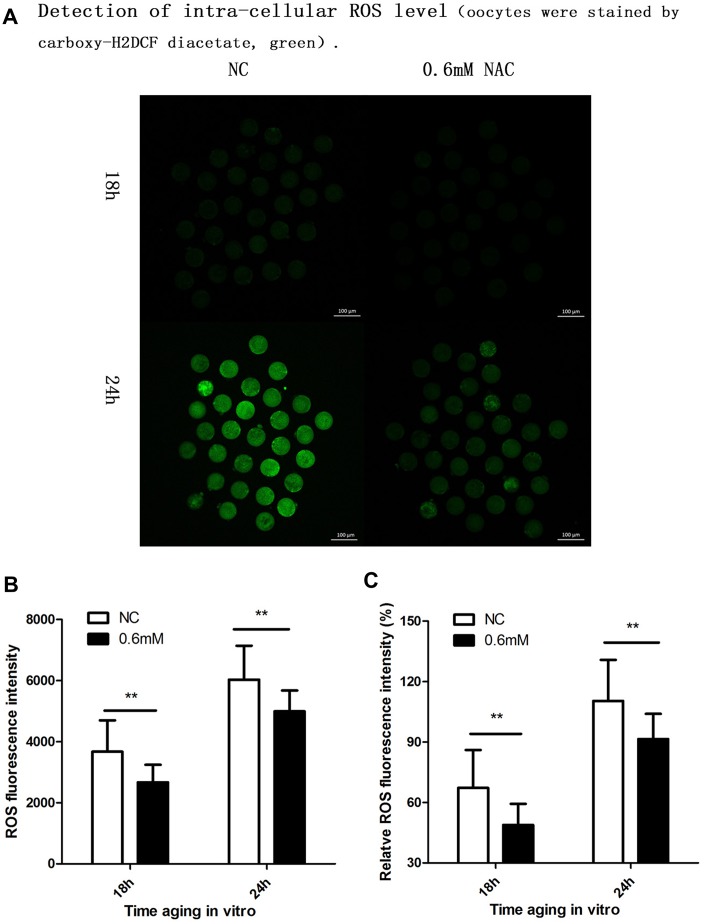
**Supplementation of NAC decreases the intra-cellular ROS level**. (**A**) Representative images of carboxy-H2DCF fluorescence in oocytes. Images were analyzed by a Perkin Elmer precisely Ultra VIEW VOX Confocal Imaging System with the identical fluorescence parameters. NC, control group in which oocytes were not treated with NAC. 0.6mM NAC, oocytes treated with 0.6mM NAC. 18h, 24h: timing of aging in vitro. (**B**) Quantitative analysis (counts of photons for each oocyte) of fluorescence intensity. The fluorescence intensity analysis for each oocyte was conducted using ZEN (2012) software. NC, control group in which oocytes were not treated with NAC. 0.6mM, oocytes treated with 0.6mM NAC. Double star represents mean difference, p < 0.01. Data are expressed as mean ± SEM of at least 3 independent experiments, and 6 superovulated mice were killed to obtain a minimum of 40 oocytes for each experiment. (**C**) ROS relative fluorescent intensity in oocytes. The fluorescence intensity of oocytes which were cultured for 24h in the control group, was set as 100%. The y-axis represents the percentage of fluorescence intensity for each group compared to the group which was set as 100%. NC, control group in which oocytes were not treated with NAC. 0.6mM NAC, oocytes treated with 0.6mM NAC. Double star represents mean difference, p < 0.01. Data are expressed as mean ± SEM of at least 3 independent experiments, and 6 superovulated mice were killed to obtain a minimum of 40 oocytes for each experiment.

Cellular increase in reactive oxygen species (ROS) level is further considered to be an important change in post-ovulatory oocyte aging. So we further investigated whether NAC treatment could protect oocyte quality by decreasing the intra-cellular ROS level. Because we found protective effects of NAC supplementation on spindle integrity and CG distribution at 18h and 24h during post-ovulatory oocyte aging in vitro, the ROS level was analyzed at 18h and 24h of culture. Oocytes collected and analyzed at 24h of post-ovulatory aging showed increased ROS levels when compared to oocytes aged for 18h. When MII oocytes were exposed to 0.6mM NAC, the ROS level observed at 18h or 24h was significantly lower compared to oocytes in the control group (Figure 4), indicating reduced ROS by NAC supplementation.

### NAC treatment protects the function of mitochondria during post-ovulatory oocyte aging in vitro

**Figure 5:**
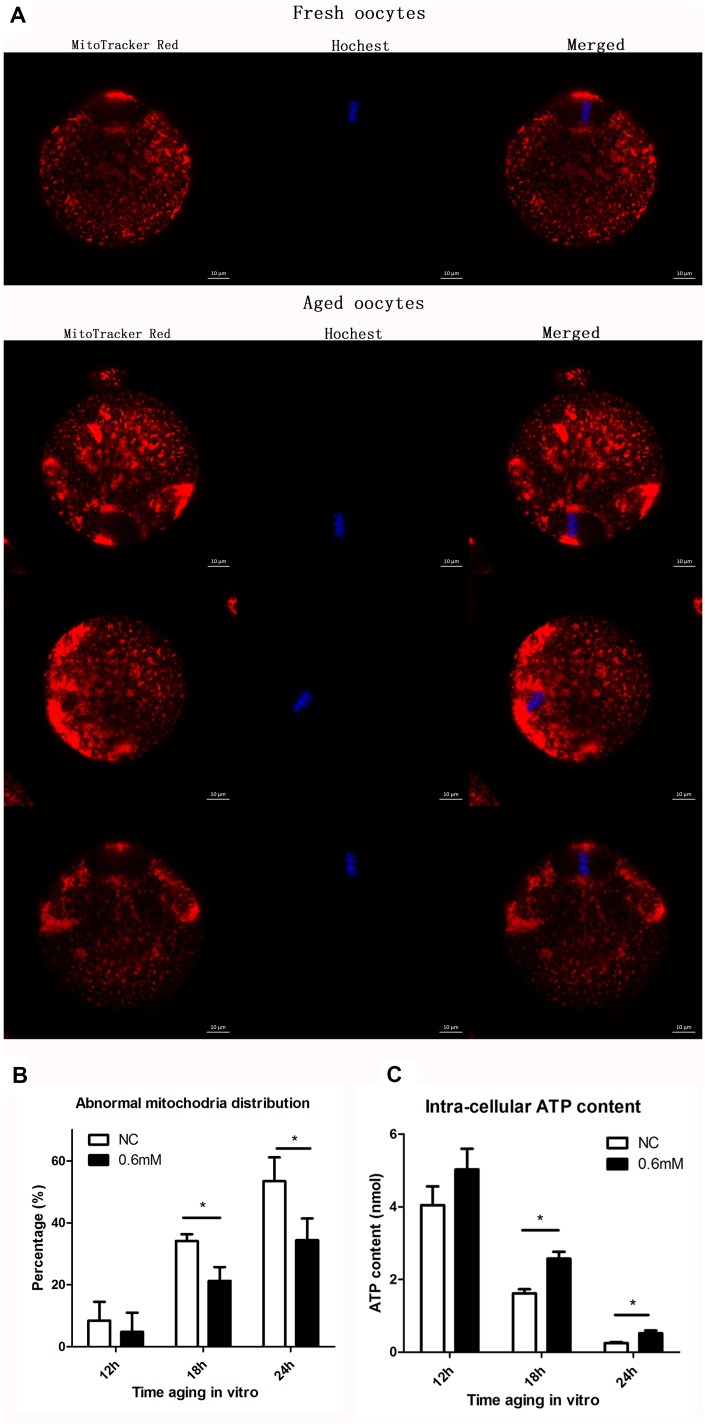
**NAC treatment protects the function of mitochondria during post-ovulatory oocyte aging in vitro.** (**A**) Confocal micrographs of mitochondrial distribution. Mitochondria were stained with MitoTracker-Red, and chromosomes were stained with Hoechst 33342 (blue). (**B**) Percentages of abnormal distribution of mitochondria in oocytes. Oocytes with normal mitochondria distribution and the those with abnormal mitochondira distribution were counted for calculating the percentage of abnormal mitochondria distribution found in oocytes in each experiment. NC, control group in which oocytes were not treated with NAC. 0.6mM, oocytes treated with 0.6mM NAC. Data are expressed as mean ± SEM of at least 3 independent experiments, and 6 superovulated mice were killed to obtain a minimum of 40 oocytes for each experiment. Star represents mean difference, 0.01< p < 0.05. (**C**) The adenosine triphosphate (ATP) content of mouse oocyte at different aging time points of two groups with or without NAC treatment. A Berthold Lumat LB 9501 luminometer and a commercial assay kit were used for ATP measurement. Six superovulated mice were killed to obtain a minimum of 40 oocytes to investigate the mitochondria distribution for each independent replicates, and 14 superovulated mice were killed to obtain a minimum of 100 oocytes to analyze the ATP content for each independent replicate.

We next examined whether the function of mitochondria could be maintained effectively with NAC supplementation. Two experiments were designed to test the function of mitochondria. The first experiment was to show the distribution of mitochondria by fluorescence staining, and the second was to analyze the cellular ATP level. Confocal microscopy showed different distribution patterns of mitochondria in oocytes aging in vitro (Figure 5A). Mitochondria of fresh oocytes displayed even distribution, referred to as normal distribution while aged oocytes showed asymmetric clustered distribution. At 18h or 24h of post-ovulatory oocyte aging, the proportion of the abnormal distribution pattern was significantly decreased in the NAC-treated group when compared to the control group (Figure 5B). Oocytes were collected at 12h, 18h and 24h of aging for ATP content analysis. No significant difference was found between control groups and treatment groups at 12h. However, the ATP level at 18h and 24h was clearly higher in NAC-treated oocytes than in oocytes without NAC treatment (Figure 5C). These results suggest that treatment with NAC during post-ovulatory oocyte aging can help to maintain normal mitochondrial functions.

## DISCUSSION

As of 2017, in couples has become a major problem, and the proportion of infertility has increased to 15%-20%. Many reasons have been attributed to causes of female infertility such as ovulation dysfunction and Fallopian tube blockage. However, post-ovulatory oocyte aging is regarded as one of the important reasons for failed fertilization and embryonic development. In this study, we investigated whether NAC, an antioxidant, has the ability to delay post-ovulatory oocyte aging in vitro, which might enhance clinical research to overcome the problem of post-ovulatory oocyte aging in humans.

Although the antioxidant NAC has already been known to alleviate oxidative stress in mitotic cells, and to improve oocyte quality of in vitro matured pig and bovine oocytes [24, 25], we still do not know whether NAC has a protective role in delaying post-ovulatory oocyte aging. Therefore, we first applied different concentrations of NAC to the M2 medium to observe its effects on post-ovulatory oocyte aging. We found that the percentage of abnormal spindles was decreased when 0.3mM, 0.6mM or 1.0mM NAC was supplemented. We also tried higher concentrations of NAC for supplementation and, unexpectedly, when the concentration was higher than 3mM, the zona pellucida of oocytes became disrupted because of the acidic environment produced by NAC (data not shown).

As the quality of oocytes decreased in a time-dependent manner compared with fresh oocytes, we mainly tested the quality of oocytes during different culture times to reflect the delay of post-ovulatory oocyte aging caused by NAC treatment. We first paid attention to the spindle changes during post-ovulatory oocyte aging in the control group, and we found that morphological changes were hardly found until aging for 12h, and the percentages of abnormal spindles rose to 33.7% and 44.7% at 18h and 24h of aging, respectively. We also analyzed the oocytes cultured for 36h in vitro, and almost all the spindles became abnormal (data not shown). When comparing the NAC treatment groups and the control groups, the percentage of abnormal spindles at 6h and 12h did not show any difference between these two groups. Notably, as shown in Figure 2B, oocyte analysis at 18 h and 24h showed that the proportion of aberrant spindles was significantly reduced in the NAC group compared to the control group (33.7 ± 3.4 vs. 23.9 ± 3.2 %, P = 0.030 < 0.05; 47.7 ± 6.6 vs. 22.9 ± 4.3 %, P = 0.026 < 0.05). We then analyzed the CG distribution pattern. As expected, differences were not found at 6h and 12h (0.7 ± 0.6 vs. 1.3 ± 0.3%, P = 0.51 > 0.05; data not shown; 7.2 ± 1.8 vs. 10.1 ± 1.3%, P = 0.09 > 0.05; Figure 3B). However, the percentage of abnormal distribution of CGs was clearly decreased at 18h and 24h in NAC-treated oocytes compared to control untreated oocytes (46.0± 5.4 vs. 32.6 ± 5.6%, P =0.03 < 0.05; 90.5 ± 4.4 vs. 76.0 ± 4.6%, P =0.01 < 0.05 Figure. 3B).

An important indicator for the quality of oocytes is the function of mitochondria. We divided the experiment into three parts to analyze whether the function of mitochondria was maintained by NAC supplementation during post-ovulatory oocyte aging. Based on the results we obtained from the analysis of spindles and CG distribution, we decided to study the time of aging at 18h and 24h; the oocytes cultured for 12h with or without treatment were set as control to show the changes. As cellular ROS was a typical indicator of post-ovulatory oocyte aging, we first tested the level of ROS during post-ovulatory aging in vitro. The raw counts we obtained for each group are shown in Figure 4B, and we also calculated the relative ROS levels to highlight the difference in ROS level for each group, which can be seen in Figure 4C. The results in Figure 4B and Figure 4C show significantly decreased levels of ROS in treated oocytes compared to those in untreated controls at 18h and 24h. Relative ROS levels calculated by the Confocal Imaging System at 18h in the control groups was 3672.83± 1014.99 for each oocyte, and it was only 2669.37 ± 1298.65 in treated groups at 18h (P value 0.009), which was decreased 27.32% compared with the fluorescent intensity number in control groups, indicating significant difference between the two groups. As for the time of 24h the relative ROS level was 6030.11 ± 1224.04 in control groups and 4993.37 ± 1257.60 in treated groups (p value 0.002); the fluorescent intensity decreased 17.19% compared with that in control groups, also indicating a significant difference. Thus, NAC treatment decreases ROS production during post-ovulatory oocyte aging in vitro.

We then analyzed the distribution of mitochondria in oocytes, and as shown in Figure 5B, the mitochondrial distribution was mostly normal after culture for 12h (8.4 ± 6.3 for control vs. 4.8 ± 7% for treatment group); oocyte analysis at 18h and 24h showed that mitochondria-defective phenotypes were clearly decreased in NAC-treated oocytes compared to control untreated oocytes (34.1 ± 2.5 vs. 21.2 ± 5.0%, P = 0.01 < 0.05; 53.4 ± 6.6 vs. 34.4 ± 8.1%, P = 0.03 < 0.05). The ATP level was also analyzed in this study, and the data in Figure 5C showed an obviously downward trend over time, which was consistent with the trend of aging reported in a previous article [6], while ATP level was maintained by supplementation with NAC in culture medium during post-ovulatory oocyte aging in vitro.

In conclusion, our research demonstrates that treatment with NAC could help to maintain high oocyte quality during post-ovulatory oocyte aging in vitro. In other words, NAC is shown to have the ability to delay post-ovulatory oocyte aging. This new finding might benefit for further research on methods aimed at delaying post-ovulatory oocyte aging and lead to applications of NAC in clinical treatments.

Antioxidants are commonly used to diminish cellular oxidative stress, and might help to delay post-ovulatory oocyte aging. Antioxidants are composed of two types: enzymatic and non-enzymatic. Enzymatic antioxidants can convert peroxides to water and alcohol; however, most non-enzymatic antioxidants work as an electron donor and therefore as a reducing agent. Some antioxidants such as Vitamin C and Vitamin E [26], as well as NAC which we used in our study, can effectively decrease intra-cellular ROS level by supplementation. We could not find any difference in oocyte morphology between treatment groups and control groups (data not shown). Other antioxidants such as quercetin and melatonin can also maintain the morphology of oocytes aged for 24 hour in vitro [27, 28]. Melatonin has also been shown to have similar effects as NAC, which can maintain mitochondrial functions during oocyte aging in vitro [28]. In this study, we also investigated whether NAC had influence on oocyte maturation, but we did not find any difference between the NAC treatment groups and the control groups (data not shown), which was similar to the function of embelin. Embelin supplementation to in vitro maturation medium did not influence nuclear and cytoplasmic maturation of pig oocytes [29]. Further study needs to be conducted to determine whether NAC treatment has beneficial effects on embryonic development.

## MATERIALS AND METHODS

All chemicals and culture media were purchased from Sigma Chemical Company (St. Louis, MO) unless stated otherwise.

### Oocyte collection

6–8 week-old ICR mice were used in this study, and they were kept in accordance with policies promulgated by the Ethics Committee of the Institute of Zoology, Chinese Academy of Sciences. To induce superovulation, female mice received intraperitoneal injection of 8 IU PMSG followed 48 h later by 8 IU hCG. The superovulated mice were killed 12-14h after hCG injection and the oviductal ampullae were broken to release the cumulus-oocyte complexes (COCs). The cumulus cells were removed by pipetting the M2 medium, containing 0.1% hyaluronidase; only denuded MII oocytes were used for the experiments. We set the time 0 hour at 12-14 h of hCG injection. Oocytes were placed in M2 medium under liquid paraffin oil at 37 °C in an atmosphere of 5% CO2 in air.

### Oocyte treatment

Collected MII oocytes were washed at least three times and immediately cultured in M2 medium supplemented with or without different concentrations of NAC (Cat #S A7250 Sigma-Aldrich). The optimal concentration of NAC was obtained from previously published studies [24, 30–32]. The fresh oocytes, which were collected after 12–14 h of hCG injection without any culture or treatment, were used as control in our study.

### Immunofluorescence microscopy

Immunofluorescent staining of spindles was performed as described previously [33]. Briefly, oocytes were fixed in 4% paraformaldehyde in PBS buffer for 30 minutes at room temperature. After being permeabilized with 0.5% Triton X-100 for 20 minutes, they were then blocked in 1% BSA-supplemented PBS for 1 hour at room temperature. For single staining of α-tubulin, oocytes were incubated overnight at 4°C with 1:800 anti-α-tubulin-FITC antibodies (Cat #2125, Beverly, MA). The method for immunofluorescent staining of CGs was performed according to a previous study [34]. Briefly, the zona pellucida was removed by treating the oocytes with Tyrode’s Solution for 1 minute. After being washed three times in a washing solution (M2 supplemented with 0.3% BSA and 0.01% TritonX-100), oocytes were fixed with 3.7% paraformaldehyde in M2 for 30 min at room temperature. The oocytes were then blocked three times (5 min each time) in a blocking solution (M2 containing 0.3% BSA and 100 mM glycine). After permeabilization for 5 min in M2 containing 0.1% Triton X-100, oocytes were washed two additional times (5 min each) in blocking solution and then cultured with 1:200 lens culinaris (LCA)-FITC for 2 hours at room temperature. The DNA was stained in the final incubation step for 15 min with Hoechst 33342. For mitochondrial staining, oocytes were incubated for 20 min at 37°C in M2 supplemented with 200 nM MitoTracker Red (Cat #M7512, Invitrogen, USA). After washing, oocytes were stained with Hoechst 33342 (10 mg/ml) for 10 min. Finally, oocytes were mounted on glass slides and viewed under a confocal laser scanning microscope (Zeiss LSM 780).

### Time-lapse live imaging experiments

After microinjecting MAP4-eGFP mRNA and H2B-mCherry mRNA, oocytes were incubated for 1h in M2 medium. Microtubule and chromosome dynamics were filmed on a Perkin Elmer precisely Ultra VIEW VOX Confocal Imaging System. Oocytes were exposed once an hour for 10h, and then exposed every 20 min for an extra 12h. The acquisition of digital time-lapse images was controlled by IP Lab (Scanalytics) or AQM6 (Andor/Kinetic-imaging) software packages. Confocal images were acquired with a 20x oil objective on a spinning disk confocal microscope.

### Detection of intra-cellular ROS level

Method for ROS level detection in oocytes was conducted as described in a previous study [34]. The procedure is briefly summarized as follows. Oocytes were collected at different aging times with or without NAC treatment, and then incubated for 30 min at 37°C in M2 supplemented with 10 mM carboxy-H2DCF diacetate (Cat #S0033, Beyotime). After washing in M2 medium for at least 3 times, oocytes were observed under a spinning disk confocal microscope (Perkin Elmer) with identical settings. Images were analyzed by a Perkin Elmer precisely Ultra VIEW VOX Confocal Imaging System. The fluorescence intensity analysis for each oocyte was conducted using ZEN (2012) software.

### Measurement of cytoplasmic ATP content in oocytes

The measurement was performed using a Berthold Lumat LB 9501 luminometer (Berthold Technologies, Bad Wildbad, Germany) and a commercial assay kit based on the luciferin–luciferase reaction (Bioluminescent Somatic Cell Assay Kit, Sigma) following the method described previously [35] following the manufacturer’s recommendations. A standard curve containing 11 ATP concentrations from 10 fmol to 10 pmol was first generated before the detection of ATP content in oocytes. For each experiment, one hundred oocytes were loaded in 1.5 ml PCR tubes, and then stored at -80°C until measurement of ATP content. The ATP content was finally calculated using the formula derived from the linear regression of the standard curve.

### Statistical analysis

For each experiment, at least 3 replicates were performed. Statistical analyses were conducted by analysis of variance. Differences between two groups were compared by using an unpaired Student’s t-test. In all analyses, data were expressed as means S.E.M, and a p < 0.05 was considered significant.
